# BAR-SH3 sorting nexins are conserved interacting proteins of Nervous wreck that organize synapses and promote neurotransmission

**DOI:** 10.1242/jcs.178699

**Published:** 2016-01-01

**Authors:** Fiona P. Ukken, Joseph J. Bruckner, Kurt L. Weir, Sarah J. Hope, Samantha L. Sison, Ryan M. Birschbach, Lawrence Hicks, Kendra L. Taylor, Erik W. Dent, Graydon B. Gonsalvez, Kate M. O'Connor-Giles

**Affiliations:** 1 Laboratory of Cell and Molecular Biology, University of Wisconsin-Madison, Madison, WI 53706, USA; 2 Cell and Molecular Biology Training Program, University of Wisconsin-Madison, Madison, WI 53706, USA; 3 Cellular Biology and Anatomy, Georgia Regents University, Augusta, GA 30912, USA; 4 Neuroscience Training Program, University of Wisconsin-Madison, Madison, WI 53705, USA; 5 Department of Neuroscience, University of Wisconsin-Madison, Madison, WI 53705, USA; 6 Laboratory of Genetics, University of Wisconsin-Madison, Madison, WI 53706, USA

**Keywords:** Synaptogenesis, Neurotransmission, *Drosophila* neuromuscular junction, Nervous wreck, Sorting nexin

## Abstract

Nervous wreck (Nwk) is a conserved F-BAR protein that attenuates synaptic growth and promotes synaptic function in *Drosophila*. In an effort to understand how Nwk carries out its dual roles, we isolated interacting proteins using mass spectrometry. We report a conserved interaction between Nwk proteins and BAR-SH3 sorting nexins, a family of membrane-binding proteins implicated in diverse intracellular trafficking processes. In mammalian cells, BAR-SH3 sorting nexins induce plasma membrane tubules that localize NWK2, consistent with a possible functional interaction during the early stages of endocytic trafficking. To study the role of BAR-SH3 sorting nexins *in vivo*, we took advantage of the lack of genetic redundancy in *Drosophila* and employed CRISPR-based genome engineering to generate null and endogenously tagged alleles of *SH3PX1*. SH3PX1 localizes to neuromuscular junctions where it regulates synaptic ultrastructure, but not synapse number. Consistently, neurotransmitter release was significantly diminished in *SH3PX1* mutants. Double-mutant and tissue-specific-rescue experiments indicate that SH3PX1 promotes neurotransmitter release presynaptically, at least in part through functional interactions with Nwk, and might act to distinguish the roles of Nwk in regulating synaptic growth and function.

## Abstract (precis)

**Summary:** A loss-of-function study of the SNX9, SNX18 and SNX33 homolog SH3PX1 in *Drosophila* reveals a new role in the development of presynaptic active zones and regulation of neurotransmitter release.

## INTRODUCTION

Synapse formation and function are crucial to the development and behavioral output of neural circuits. Genetic studies in *Drosophila* have identified Nervous wreck (Nwk) as a central regulator of synaptic growth and physiology. Loss of Nwk results in ectopic synapse formation and severely diminished neurotransmitter release at the larval neuromuscular junction (NMJ), indicating that Nwk functions to both attenuate synaptic growth and promote synaptic function (Coyle et al., 2004). With their combination of membrane- and protein-binding domains, Nwk-family proteins are well suited to regulate intracellular protein trafficking. Understanding their function could provide insights into how intracellular modulators of signaling pathways coordinate and distinguish the many signals impinging on developing neurons.

Nwk is a conserved member of the F-BAR family of membrane-binding proteins, which has primarily been associated with endocytosis and vesicle trafficking (Futterer and Machesky, 2007; Itoh et al., 2005). The *Drosophila* genome encodes a single Nwk family member, whereas mammalian genomes encode two Nwk proteins, NWK1/FCHSD2 and NWK2/FCHSD1 (Coyle et al., 2004). F-BAR and closely related BAR domains are unique membrane-binding modules that form crescent-shaped homodimers that bind to and generate curved membranes (Daumke et al., 2014; Frost et al., 2009; Itoh et al., 2005; Suetsugu et al., 2010). Nwk proteins also contain two SH3 domains and a C-terminal proline-rich domain with numerous SH3-binding sites, which together mediate multiple interactions with endocytic, cytoskeletal and signaling proteins (Coyle et al., 2004; O'Connor-Giles et al., 2008; Rodal et al., 2011, 2008). Studies of the *in vivo* function of Nwk have been conducted in *Drosophila*. These studies have focused on Nwk-dependent attenuation of NMJ growth and have demonstrated that Nwk negatively regulates a retrograde (muscle to neuron) BMP growth signal in concert with endocytic proteins Dap160/intersectin, Shibire/dynamin, Endophilin and Sorting Nexin 16 (Snx16) (Coyle et al., 2004; O'Connor-Giles et al., 2008; Rodal et al., 2011, 2008). Genetic interactions with the Wingless/Wnt signaling pathway indicate that the role of Nwk in the intracellular modulation of growth signaling at the NMJ extends to multiple pathways (Rodal et al., 2011).

In contrast, the pathway or pathways through which Nwk promotes neurotransmitter release are unknown. Ultrastructural, electrophysiological and dye-uptake assays indicate that synaptic vesicle cycling is normal, and point instead to developmental defects (Coyle et al., 2004). To identify conserved proteins that might distinguish the role of Nwk in regulating synapse number and function, we isolated mammalian Nwk interactors through mass spectrometry. Here, we detail the identification of a conserved interaction between Nwk and BAR-SH3 sorting nexins – a subgroup of the large and broadly conserved sorting nexin family of phosphoinositide-binding proteins that utilizes its distinct membrane-binding domains to promote multiple cellular events, including clathrin-mediated endocytosis (Cullen, 2008; Haft et al., 1998; Lundmark and Carlsson, 2009). Through co-immunoprecipitation experiments, we demonstrate interactions between NWK2 and all three mammalian BAR-SH3 sorting-nexin-family members. We find that the interaction is conserved between Nwk and SH3PX1, the sole *Drosophila* representative of the BAR-SH3 sorting nexin family.

To study the *in vivo* role of SH3PX1, we turned to the *Drosophila* nervous system where functional studies are not complicated by genetic redundancy, and where Nwk and other proteins known to interact with BAR-SH3 sorting nexins have roles in synapse development and neuronal function (Coyle et al., 2004; Dickman et al., 2006; Poodry and Edgar, 1979). Through CRISPR-based genome engineering, we generated endogenously tagged and null alleles of *SH3PX1*. At the *Drosophila* NMJ, SH3PX1 localized to the plasma membrane of both postsynaptic and presynaptic terminals, where it overlapped with Nwk. Synapse number was normal in *SH3PX1* mutants, indicating that Nwk modulates growth signals independently of SH3PX1. In contrast, neurotransmitter release was significantly disrupted by the loss of SH3PX1. Rescue and double-mutant analyses suggested that SH3PX1 and Nwk regulate presynaptic function, at least in part, through a common pathway. Although synaptic vesicle biogenesis was largely normal, ultrastructural analyses revealed significant abnormalities at *SH3PX1*-mutant active zones, consistent with an underlying developmental cause for the deficit in neurotransmitter release. Taken together, our findings identify a new requirement for BAR-SH3 sorting nexins in organizing the establishment of functional synapses and indicate that SH3PX1 could enable Nwk to differentially modulate multiple signaling pathways to achieve diverse outcomes.

## RESULTS

As Nwk contains multiple protein–protein-interaction domains through which it interacts with signaling molecules and key components of intracellular trafficking pathways (Coyle et al., 2004; O'Connor-Giles et al., 2008; Rodal et al., 2011, 2008), we reasoned that a ‘guilt-by-association’ approach might identify previously unknown Nwk-interacting proteins that shed light on how Nwk coordinates its diverse roles. We further reasoned that selecting mammalian Nwk as a starting point would facilitate the identification of evolutionarily conserved interacting proteins that might function in neurotransmission – a hypothesis we could test *in vivo* in *Drosophila*.

### NWK2 is expressed in cortical neurons during synaptogenesis

**Fig. 1.**
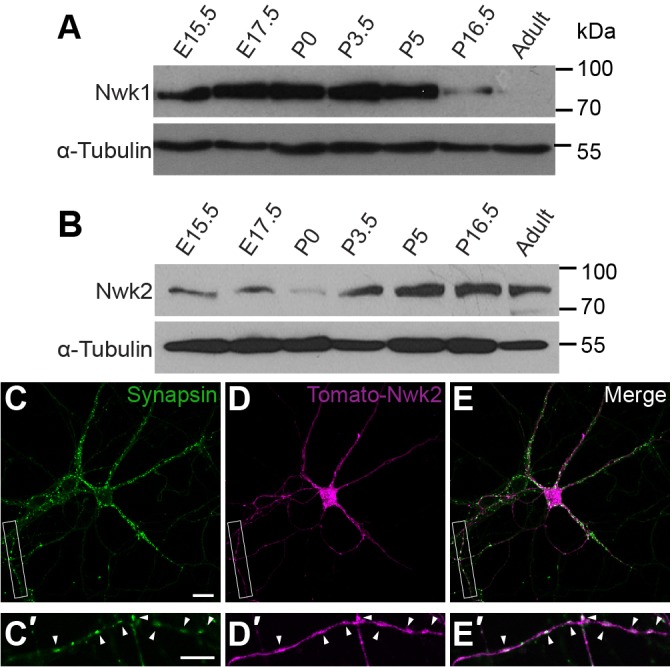
**NWK2 is expressed in cortical neurons during synaptogenesis.** (A,B) Temporal expression of NWK1 and NWK2 in mouse cerebral cortex during embryonic (‘E’) stages through to adult. NWK1 expression declines at 16.5 days of postnatal development (P16.5) and is substantially reduced in adulthood, whereas NWK2 expression is maintained through development. Equal loading was confirmed by assessing levels of α-tubulin. (C–E′) Cortical neurons at 12 days *in vitro* after transfection with NWK2–Tomato (magenta) and stained for the presynaptic marker synapsin (green). (C′–E′) Higher magnification of the boxed regions in C–E. Arrowheads indicate colocalization. Scale bars: 20 µm (C–E); 10 µm (C′–E′).

As a first step toward identifying conserved pathways through which Nwk might promote the development of properly functioning synapses, we sought to determine whether mammalian NWK1 and/or NWK2 are expressed in the nervous system during synaptogenesis by analyzing developmentally staged tissue from the mouse cerebral cortex. Both NWK1 and NWK2 are expressed in the mouse brain during embryogenesis and postnatal development (Fig. 1A,B). Notably, NWK1 expression declined significantly by postnatal day 16.5, a period of dramatic increase in synapse number. In contrast, NWK2 expression was maintained throughout postnatal development and into adulthood. Thus, similar to *Drosophila* Nwk (Coyle et al., 2004), mammalian NWK2 is highly expressed in the central nervous system (CNS) during postnatal periods of synaptogenesis, consistent with a role in synapse development.

*Drosophila* Nwk is neuron specific and broadly expressed throughout the nervous system. At the larval NMJ, where presynaptic boutons and their postsynaptic counterparts can be visualized in detail, Nwk localizes to the periactive zone of presynaptic boutons, a specialized membrane region, notable for the presence of numerous endocytic proteins, where both synaptic vesicle endocytosis and regulatory signaling occur (Coyle et al., 2004; Koh et al., 2004; Marie et al., 2004; Sone et al., 2000). To investigate NWK2 localization in mammalian neurons, we expressed full-length td-Tomato-tagged NWK2 in cultured cortical neurons. At 12 days *in vitro*, we observed broad expression throughout neurons (Fig. 1C–E). Discrete regions of overlap with the synaptic vesicle protein synapsin indicated that NWK2 is present at presynaptic terminals (Fig. 1C′–E′).

### NWK2 interacts with sorting nexins 9, 18 and 33

NWK2 expression in the mouse CNS is consistent with the possibility of a conserved role in synapse development and function, so we conducted liquid-chromatography-tandem mass spectrometry (LC-MS/MS) to identify NWK2-interacting proteins. FLAG–NWK2 was transiently expressed in the CAD (Cath.a-differentiated) murine CNS cell line (Qi et al., 1997). Following affinity purification of interacting protein complexes and in-solution trypsin digestion, soluble fractions were subjected to high-resolution LC-MS/MS. To obtain a high-confidence set of conserved NWK2-interacting proteins, we considered spectra with greater than 95% confidence. We excluded (1) proteins represented in a parallel control with untransfected CAD lysates, (2) proteins identified by only one peptide, and (3) common contaminants, including ribosomal and mitochondrial proteins, and proteins previously identified as non-specific binders of agarose beads (Mellacheruvu et al., 2013; Trinkle-Mulcahy et al., 2008). Eleven proteins met these criteria, eight of which have clear homologs in *Drosophila* – all of which are expressed in neurons (Table 1).

**Table 1.**
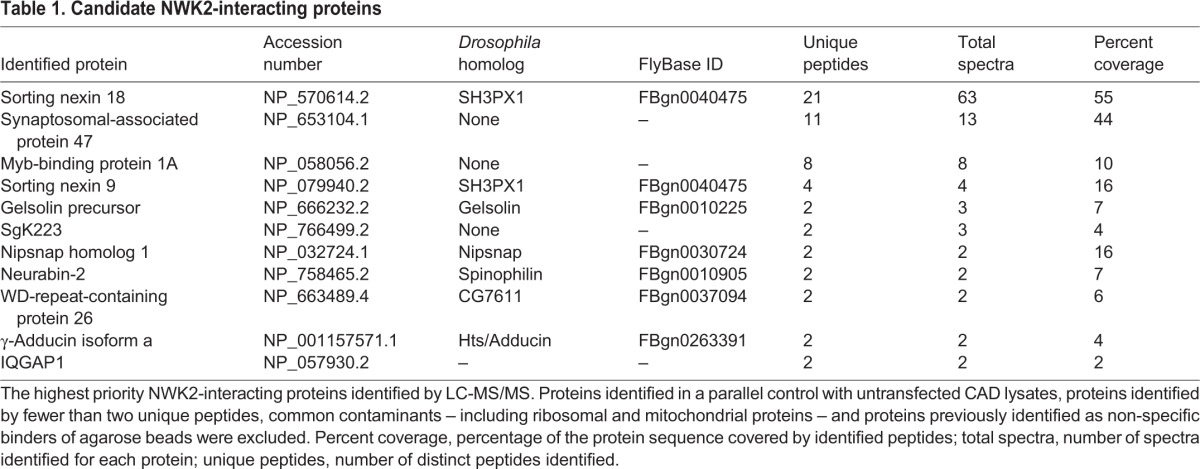
**Candidate NWK2-interacting proteins**

**Fig. 2.**
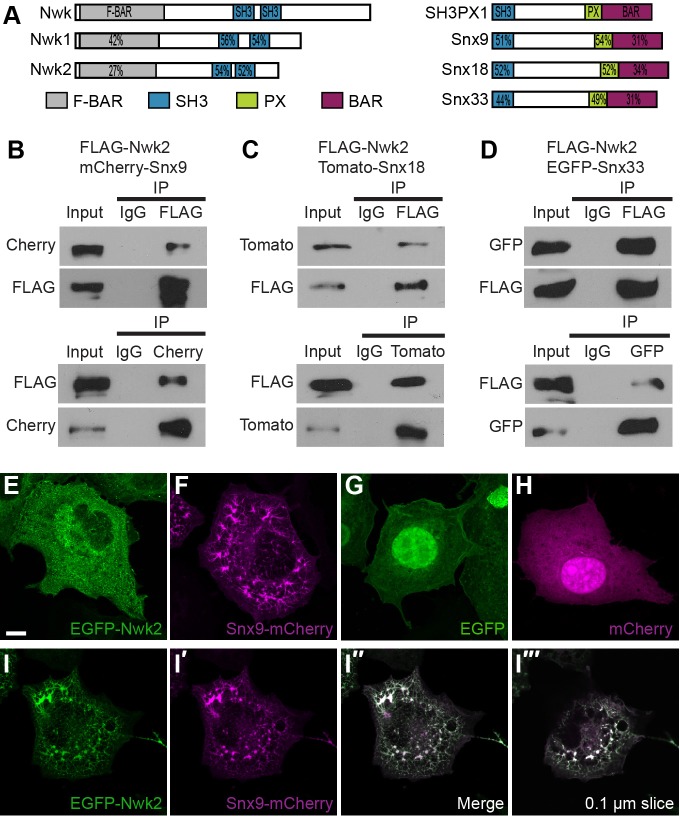
**NWK2 physically interacts with SNX9, SNX18 and SNX33, and localizes to tubules induced by BAR-SH3 sorting nexins.** (A) Domain structure of Nwk and BAR–SH3 sorting nexins in *Drosophila* and mouse. Percent identity between *Drosophila* and each mouse protein is indicated for the conserved domains. (B–D) Co-immunoprecipitation experiments between NWK2 and BAR-SH3 sorting nexins. COS-7 cells were transfected with FLAG–NWK1 and mCherry–SNX9 (B), Tomato–SNX18 (C) or EGFP–SNX33 (D). Immunoprecipitation was performed with an antibody against FLAG (upper panel) and confirmed with reciprocal immunoprecipitation experiments with antibodies against the appropriate fluorescent tag (lower panel). Input lanes contain lysate equal to one-fifth of the amount used for the pull-down assays. IP indicates the antibody used for immunoprecipitation. (E–H) COS-7 cells that had been transfected with GFP–NWK2 (E), mCherry–SNX9 (F), GFP (G) or mCherry (H) alone. Considerable tubule formation is observed upon overexpression of SNX9. (I–I‴) Co-transfection of EGFP–NWK2 and SNX9 resulted in the redistribution of NWK2 to tubules induced by SNX9. Scale bar: 10 µm.

Two interactors, sorting nexin 18 (SNX18) and the related sorting nexin 9 (SNX9), immediately caught our attention for their roles in endocytosis and intracellular trafficking, and thus, potential to function with Nwk proteins in coordinating intracellular signaling (Cullen, 2008; Haft et al., 1998). Sorting nexins are a conserved family of membrane-binding proteins with over 30 members in mammals and eight in *Drosophila*. Sorting nexins are related by the presence of a phosphoinositide-binding PX domain, and further subclassified by the presence of additional domains that mediate their diverse cellular roles in membrane trafficking. SNX9 and SNX18 belong to the BAR-SH3 subfamily, along with SNX33 (Lundmark and Carlsson, 2009). BAR-SH3 sorting nexins contain an N-terminal SH3 domain and C-terminal BAR domain in addition to the defining PX domain (Fig. 2A). Through these domains, SNX9, the most-studied family member, binds to highly curved phosphatidylinositol-4,5-bisphosphate-enriched plasma membrane subdomains where it interacts with dynamin and WASP to promote endocytosis (Lundmark and Carlsson, 2009). SNX18 has functions in clathrin-independent endocytosis and autophagosome biogenesis, and all three have been implicated in mitosis (Knaevelsrud et al., 2013b; Ma and Chircop, 2012; Zhang et al., 2009). A previous study also reported a physical interaction between SNX9 and NWK2, and colocalization in the mouse cochlear sensory hair cells (Cao et al., 2013), lending support to the identification of the BAR-SH3 family as biologically significant interacting proteins of NWK2.

To confirm and extend the identification of SNX9 and SNX18 as NWK2-interacting proteins, we performed co-immunoprecipitation experiments between NWK2 and each member of the BAR-SH3 family in COS-7 cells. We found that NWK2 interacts with both SNX9 and SNX18, as well as the third BAR-SH3 sorting nexin, SNX33. All three interactions were also observed in reciprocal co-immunoprecipitation experiments (Fig. 2B–D). Consistent with the finding of Cao et al. (2013), we found that the interaction with SNX9 is specific to NWK2 and mediated by the NWK2 F-BAR and SNX9 SH3 domains (Fig. S1; data not shown).

### NWK2 localizes to SNX9-, SNX18- and SNX33-induced membrane tubules

Through homophilic interactions between their BAR and F-BAR domains, both BAR-SH3 sorting nexins and Nwk-family proteins form crescent-shaped dimers capable of binding to and sculpting membranes (Daumke et al., 2014; Frost et al., 2009; Gallop and McMahon, 2005). Banana-shaped BAR domains are associated with highly curved membranes, such as the neck of budding vesicles, whereas shallower F-BAR domains are associated with membranes of lower curvature, such as nascent plasma membrane invaginations and endosomes. Overexpression of BAR proteins, including BAR-SH3-family members, induces extensive plasma membrane invaginations or tubules, demonstrating their ability to invaginate membranes (Haberg et al., 2008; Shin et al., 2008; van Weering et al., 2012). In a recent study, Becalska et al. (2013) observed membrane protrusions upon overexpression of the F-BAR domains of NWK1 and NWK2 in HEK293T cells. Coupled with their *in vivo* role in endocytosis, this suggests that Nwk proteins can participate in diverse membrane-shaping activities that might be regulated by the presence of specific binding partners.

To assess the membrane-shaping activities of full-length Nwk proteins in a cellular context, we expressed NWK2 in COS-7 cells. NWK2 expression induced weak formation of tubules, whereas expression of SNX9, SNX18 or SNX33 induced robust tubulation, as previously demonstrated (Fig. 2E–H; Fig. S2A–C) (Haberg et al., 2008; Shin et al., 2007). When co-expressed with NWK2, BAR-SH3-family members maintained the ability to induce membrane tubules. Remarkably, NWK2 colocalized with each BAR-SH3-family member at the newly formed membrane invaginations (Fig. 2I; Fig. S2D,E). NWK2 interacts with SNX9 through its F-BAR domain (Cao et al., 2013), so the binding of sorting nexins could render this domain unavailable for membrane interactions, enabling it to associate with Snx-induced tubules, despite the distinct membrane-sensing properties of the two proteins.

### Interactions between Nwk and BAR-SH3 sorting nexins are conserved in *Drosophila*

**Fig. 3.**
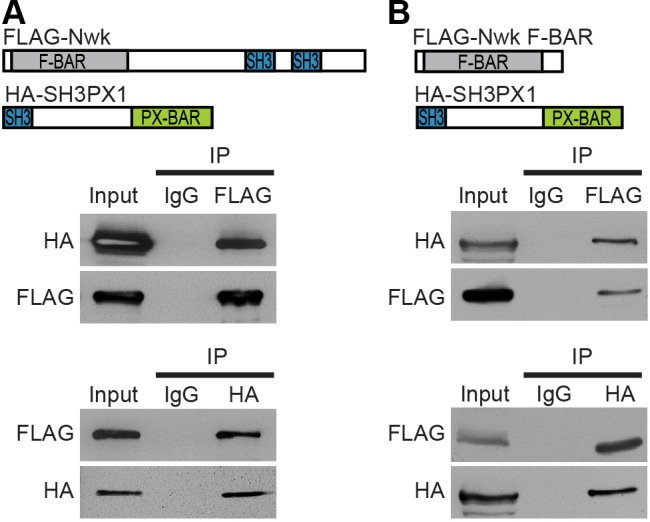
**Nwk physically interacts with the *Drosophila* BAR-SH3 protein SH3PX1.** (A,B) Co-immunoprecipitation experiments between Nwk and SH3PX1. S2 cells were transfected with HA–SH3PX1 and FLAG–Nwk (A) or FLAG–Nwk F-Bar (B). Immunoprecipitation was performed using antibodies against either FLAG (upper panel) or HA (lower panel). Input lanes contain lysate equal to one-fifth of the amount used for the pull-down assays. IP indicates the antibody used for immunoprecipitation.

To determine if Nwk–BAR-SH3-family interactions are evolutionarily conserved, we conducted co-immunoprecipitation experiments in *Drosophila* S2 cells. The *Drosophila* genome encodes a single homolog of Snx9, Snx18 and Snx33, previously named SH3PX1 for the presence of the SH3 and PX domains common to BAR-SH3 sorting nexins (Worby et al., 2002, 2001). Like its mammalian homologs, SH3PX1 also has a BAR domain. In co-immunoprecipitation experiments, we observed binding between full-length Nwk and SH3PX1 (Fig. 3A). These results were confirmed in reciprocal co-immunoprecipitation analyses, indicating that the interaction with the BAR-SH3 family is indeed conserved between mammals and *Drosophila* (Fig. 3A).

As noted, the NWK2–SNX9 interaction occurs through the NWK2 F-BAR domain and the SNX9 SH3 domain, possibly through a PxxP SH3-binding site in the F-BAR domain of NWK2 (Cao et al., 2013). To determine whether Nwk, which also contains a PxxP site in its F-BAR domain, interacts with SH3PX1 through a similar mechanism, we expressed the Nwk F-BAR domain in S2 cells and, through reciprocal co-immunoprecipitation experiments, found that it bound to SH3PX1 (Fig. 3B). Thus, the interactions between mammalian NWK2 and SNX9, SNX18 and SNX33 indeed predicted a conserved interaction between *Drosophila* Nwk and SH3PX1, opening the door to *in vivo* studies of the functional significance of the interaction at the *Drosophila* larval NMJ, where Nwk regulates synaptic growth and function (Coyle et al., 2004).

### SH3PX1 is pre- and post-synaptic at the NMJ, and colocalizes with Nwk

The *Drosophila* NMJ is a well-characterized model for the *in vivo* study of the pathways that regulate synaptic development. Motorneurons form stereotyped connections with target muscles that contain a consistent number of boutons and synapses. Each bouton contains 10–15 presynaptic active zones surrounded by a membranous periactive zone. On the muscle side, a postsynaptic density comprising glutamate receptor clusters forms opposite presynaptic terminals, and a highly specialized membrane compartment called the subsynaptic reticulum envelops the bouton. The coordinated formation of each of these components is crucial to synaptic function, and genetic studies have revealed multiple conserved pathways that control their development (Collins and DiAntonio, 2007; Harris and Littleton, 2015; Menon et al., 2013).

**Fig. 4.**
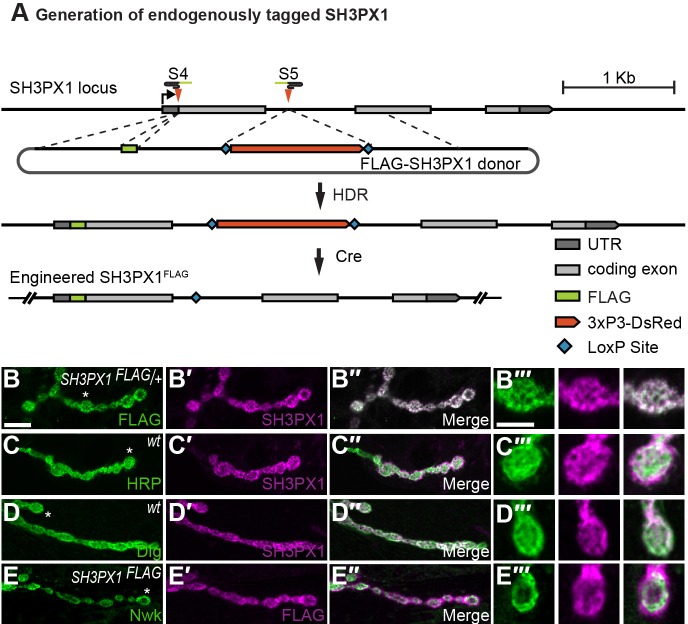
**SH3PX1 localization at the *Drosophila* NMJ.** (A) Generation of the endogenously tagged *SH3PX1^FLAG^* allele. Schematic of the *SH3PX1* locus and the HDR strategy used to insert a 3×-FLAG tag and linker at the translation start site of *SH3PX1*. S4 and S5 represent the gRNA target sites used to generate the allele. The removable 3xP3-DsRed cassette serves as a marker for identification of edited flies. (B–B″) Confocal images of flies heterozygous for endogenously FLAG-tagged *SH3PX1* (*SH3PX1^FLAG^*) co-labeled with a mouse antibody against FLAG (green) and a rabbit antibody against SH3PX1 (magenta). (C–C″) Wild-type boutons that had been co-labeled with the neuronal membrane marker HRP (green) and an antibody against SH3PX1 (magenta). (D–D″) Wild-type boutons that had been co-labeled for the predominantly postsynaptic membrane marker Dlg (green, mouse antibody) and SH3PX1 (magenta). (E–E″) *SH3PX1^FLAG^* NMJs co-labeled for Nwk (green, rabbit antibody) and FLAG (magenta), showing that staining of SH3PX1 overlaps with that of Nwk. (B‴–E‴) Higher magnification of a single bouton from each preparation. All images are of a single 0.1-µm optical plane. Asterisks indicate the boutons that are shown at higher magnification. Scale bars: 20 µm (B–E″), 5 µm (B‴–E‴).

Genome-wide studies indicate that SH3PX1 is transcribed broadly in flies both spatially and temporally, with substantial expression in the nervous system (Chintapalli et al., 2007; The modENCODE Consortium, 2010). To determine the subcellular localization of SH3PX1 in neurons, we employed CRISPR-based genome engineering to insert a 3×-FLAG tag into the *SH3PX1* locus (Fig. 4A; Table S1). Endogenously tagged SH3PX1 perfectly colocalized with SH3PX1 that had been labeled with a highly specific rabbit polyclonal antibody, demonstrating that FLAG–SH3PX1 maintains the endogenous SH3PX1 expression pattern (Fig. 4B) (Hicks et al., 2015). In the larval nervous system, SH3PX1 is expressed in neuronal cell bodies, the synaptic neuropil of the larval ventral ganglion and at motor neuron synapses (data not shown; Fig. 4B–E). At presynaptic terminals, SH3PX1 colocalized with horseradish peroxidase (HRP), which exclusively labels neuronal membranes (Fig. 4C). This is consistent with a previous demonstration of Snx9 localization to presynaptic terminals in cultured hippocampal neurons (Shin et al., 2007). In muscle, SH3PX1 localized to the subsynaptic reticulum, as evidenced by its colocalization with Discs large, a major component of the postsynapse (Fig. 4D). We next investigated the colocalization of SH3PX1 and Nwk at presynaptic terminals. At the NMJ, Nwk localizes to the honeycomb-shaped periactive zone (Coyle et al., 2004). Consistent with their conserved ability to bind to each other, we observed regions of overlap between SH3PX1 and Nwk at the presynaptic membrane (Fig. 4E).

### SH3PX1 does not restrict synaptic growth

To determine the *in vivo* function of SH3PX1 at synapses, we generated null alleles in *Drosophila*, in which the presence of only one BAR-SH3 sorting nexin facilities loss-of-function studies. We employed CRISPR-mediated homology-directed repair (HDR) to replace the endogenous *SH3PX1* locus with a visible marker (Fig. S3, Table S1) (Gratz et al., 2014). As homozygotes or over a deficiency, two independently generated *SH3PX1*-null alleles were viable with impaired fertility.

**Fig. 5.**
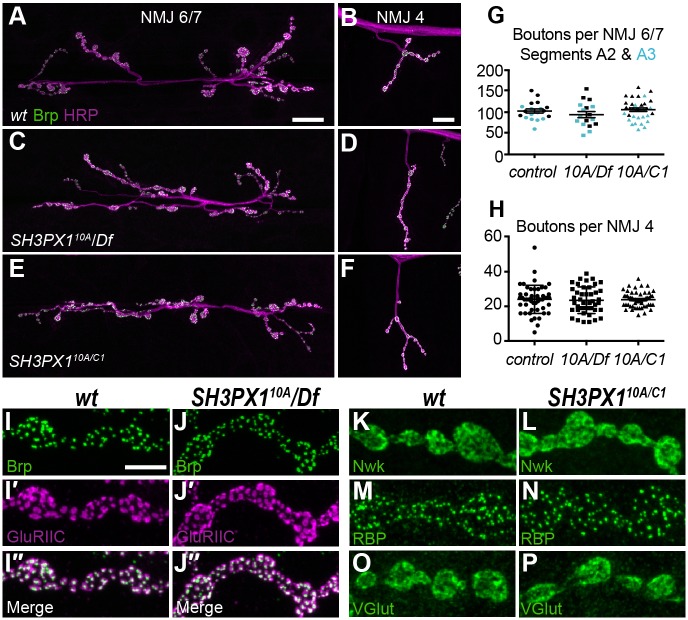
**Synaptic growth is not altered in *SH3PX1* mutants.** (A–F) Confocal images of NMJ 6/7 (A,C,E) and NMJ 4 (B,D,F) preparations labeled for Bruchpilot (green) and Cy3-conjugated HRP in control (A,B), *SH3PX1^10A^/Df(3L)BSC392* (C,D; *10A/Df*) and *SH3PX1^10A^/SH3PX1^C1^* (E,F; *10A/C1*) larvae. (G,H) Scatter plots of the number of boutons in control and *SH3PX1* mutants at NMJ 6/7, segments A2 and A3 (G), and NMJ 4 (H). Control: *n*=19 NMJ 6/7, 50 NMJ 4; *SH3PX1^10A^/Df(3L)BSC392*: *n*=17 NMJ 6/7, 43 NMJ 4; *SH3PX1^10A^/SH3PX1^C1^*: *n*=20 NMJ 6/7, 53 NMJ 4. n.s., not significant compared to control; ANOVA followed by post-hoc tests with Šidák correction. Error bars represent s.e.m., line represents mean. (I–J″) Confocal images of control and *SH3PX1^10A^/Df(3L)BSC392* boutons labeled with antibodies against Bruchpilot (I,J) and GluRIIC (I′,J′); (I″,J″) merged images of Bruchpilot and GluRIIC labelling. (K–P) Confocal images of control and *SH3PX1^10A^/SH3PX1^C1^* boutons labeled with antibodies against Nwk (K,L), active zone cytomatrix component Rim-binding Protein (RBP; M,N) and the vesicular glutamate transporter VGlut (O,P). *wt*, wild type. Scale bars: 20 µm (A–F), 5 µm (I–P).

We assessed synaptic growth in *SH3PX1^10A^/Df(3L)BSC392* and *SH3PX1^10A^/SH3PX1^C1^* null mutants by counting boutons at two well-studied synapses, NMJ 6/7 and NMJ 4. NMJ 6/7 comprises two motor neurons that innervate near the cleft between muscles 6 and 7, and NMJ 4 is a small synapse formed by motor neuron 4-1b on the face of muscle 4 (Hoang and Chiba, 2001). NMJs of control and mutant third instar larvae were stained with HRP to label neuronal membranes and for ELKS-family homolog Bruchpilot to label active zones. We observed no difference in either total bouton or satellite bouton number between control and *SH3PX1* mutant larvae at NMJ 6/7 or NMJ 4 (Fig. 5A–H). We also quantified the number of active zones by counting Bruchpilot puncta at NMJ 4 and did not find a difference in *SH3PX1* mutants (Fig. 5I,J; control: 10.28±0.39 active zones/bouton, *n*=10 NMJs vs *SH3PX1^10A^/Df*: 10.78±0.45, *n*=10 NMJs; mean±s.e.m.). Thus, at the levels of bouton and active zone number, synaptic growth proceeds normally without SH3PX1.

We next assessed the molecular structure of synapses in *SH3PX1* mutants. A crucial aspect of synapse structure is the accumulation of postsynaptic density proteins in direct apposition to active zones in order to facilitate the rapid transfer of information. At the *Drosophila* NMJ, glutamate receptors comprising four heteromeric subunits cluster at postsynaptic densities. We co-labeled control and *SH3PX1* NMJs for Bruchpilot and the obligate glutamate receptor subunit GluRIIC, and found that their apposition is normal in *SH3PX1* mutants (Fig. 5I,J). Finally, we assessed the localization of Nwk, the active zone cytomatrix component Rim-binding Protein and the vesicular glutamate transporter VGlut at *SH3PX1* synapses, and found that each localized normally (Fig. 5K–P).

### SH3PX1 regulates active zone ultrastructure

**Fig. 6.**
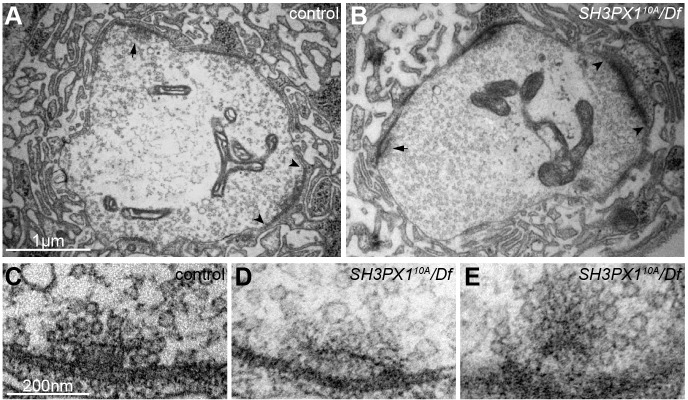
**Disrupted ultrastructure at *SH3PX1* active zones.** (A,B) Transmission electron micrographs showing midline cross sections of type Ib boutons in control (A) and *SH3PX1^10A^/Df(3L)BSC392* (B) larvae. (C–E) Higher magnification images of a typical T-bar structure at control active zones (C) and aberrant T-bar structure at *SH3PX1^10A^/Df(3L)BSC392* active zones (D,E). Instead of the typical T-bar-shaped electron density, the densities at *SH3PX1* active zones are often misshapen and/or separated from the membrane. 54 control and 86 *SH3PX1^10A^/Df(3L)BSC392* active zones from three animals per genotype were analyzed. Arrowheads indicate active zone limits, arrows point to T-bars. Scale bars: 1 µm (A,B); 200 nm (C–E).

Given that their small size puts them below the diffraction limit of light, defects in synaptic structure are often undetectable using light-level analyses. To investigate in greater detail, we examined control and *SH3PX1-*mutant larval NMJs with electron microscopy (Fig. 6A,B). Consistent with our light-level analysis, active zone density was the same in the two genotypes (control: 0.51±0.05 active zones/μm bouton perimeter, *n*=7 boutons vs *SH3PX1^10A^/Df*: 0.50±0.06, *n*=7 boutons; mean±s.e.m.). Active zone length was also normal (control: 613±41 nm, *n*=29 active zones vs *SH3PX1^10A^/Df*: 632±30 nm, *n*=34 active zones), indicating that neither synapse number nor size are regulated by SH3PX1. We also investigated the number of synaptic vesicles at control and mutant synapses by quantifying vesicles within 200 nm of active zone membranes, and found no difference (control: 20.8±1.4 vesicles per synapse, *n*=22 synapses vs *SH3PX1^10A^/Df*: 19.5±1.5 vesicles per synapse, *n*=20 synapses). Finally, we examined the size of synaptic vesicles and found a 10% increase in vesicle diameter at *SH3PX1*-mutant synapses (control: 33.45±0.39 nm, *n*=292 vesicles vs *SH3PX1^10A^/Df*: 36.95±0.40 vesicles per synapse, *n*=266 vesicles). These results suggest that SH3PX1 does not control the transport or endocytic processes that determine synaptic vesicle number at active zones, but that it might contribute to determining synaptic vesicle size.

Electron–dense projections are a prominent feature of active zones in electron micrographs. These electron-dense projections contain cytomatrix proteins that function in the clustering of readily releasable synaptic vesicles in close proximity to Ca^2+^ channels in order to coordinate synchronized release (Bruckner et al., 2015; Wichmann and Sigrist, 2010). Thus, the proper formation of dense projections is a key determinant of neurotransmitter release at active zones. At the *Drosophila* NMJ, wild-type active zones exhibit dense projections with a characteristic T-bar shape (Fig. 6C). We observed similar percentages of control and mutant active zones with electron densities (52% in control, *n*=54 active zones vs 34% in *SH3PX1^10A^/Df*, *n*=86 active zones); however, these structures were frequently malformed in *SH3PX1* mutants (Fig. 6D,E). We found that 74% of control active zones with electron-dense projections had T-bars, whereas such regularly shaped densities were only observed in 38% of *SH3PX1* active zones that exhibited electron-dense projections (*P*=0.007, chi-square test; *n*=27 control and 29 *SH3PX1* active zones with single electron-dense projections). This indicates that SH3PX1 function is required for the proper structure of a crucial organizing component of presynaptic terminals and that it might play a role in establishing normal synaptic function at the NMJ.

### SH3PX1 promotes neurotransmitter release

**Fig. 7.**
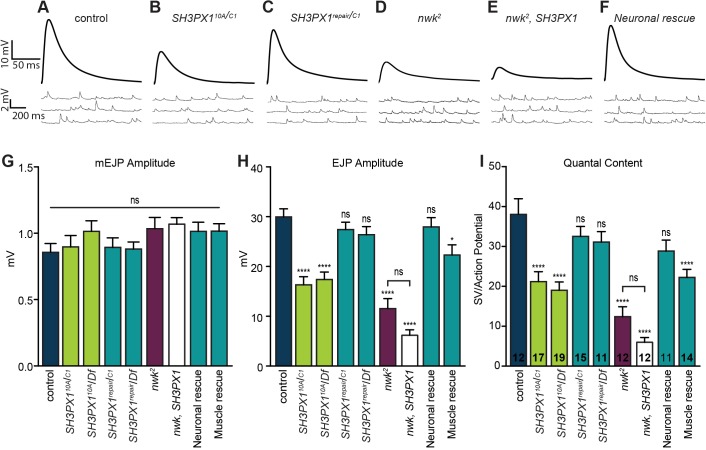
**SH3PX1 promotes neurotransmitter release.** (A–F) Representative traces of EJPs and mEJPs in control (A), *SH3PX1^10A^/SH3PX1^C1^* (B), *SH3PX1^repair^/SH3PX1^C1^* (C), *nwk^2^* (D), *nwk^2^, SH3PX1^10A^/nwk^2^, SH3PX1^C1^* (*nwk, SH3PX1*; E) and *C155-Gal4*/Y; *UAS-FLAG-SH3PX1*, *SH3PX1^10A^/SH3PX1^C1^* (neuronal rescue; F) larvae. Stimulus artifacts have been removed from representative EJPs for clarity. (G–I) Average mEJP amplitude (G), EJP amplitude (H) and quantal content (I) of larvae with the genotypes depicted in A–F and the genotypes *SH3PX1^10A^/Df(3L)BSC392*, *SH3PX1^repair^/Df(3L)BSC392*, and 24B-Gal4, *SH3PX1^C1^/*UAS-FLAG-SH3PX1, *SH3PX1^10A^*. The number of NMJs analyzed is noted for each genotype on the bars in I. n.s., not significant; *P<0.05; ****P<0.0001 compared to control; ANOVA followed by post-hoc tests with Šidák correction. Error bars represent s.e.m. mEJP amplitude was not altered in any genotype. EJP amplitude and quantal content were significantly diminished in *SH3PX1* mutants and restored to control levels upon repair of the locus or presynaptic, but not postsynaptic, expression of FLAG–SH3PX1. EJP amplitude and quantal content in *nwk SH3PX1* double mutants were not significantly different from those of *nwk* mutants.

To determine whether the altered active zone structure of *SH3PX1*-mutant terminals is accompanied by a corresponding deficit in neurotransmission, we measured spontaneous and evoked synaptic potentials through intracellular recordings at control and *SH3PX1* NMJs. Loss of *SH3PX1* did not significantly affect miniature excitatory junction potential (mEJP) amplitude, despite the mildly enlarged vesicles that we observed in electron micrographs, suggesting that these vesicles do not contain more neurotransmitter or are not spontaneously released (Fig. 7A,B,G). However, evoked excitatory junction potentials (EJPs) were severely reduced (Fig. 7A,B,H). From these data, we calculated quantal content, or the number of synaptic vesicles released per action potential, and found that neurotransmitter release decreased by approximately 50% in the absence of *SH3PX1* (Fig. 7A,B,I). Quantal content was restored to wild-type levels upon repair of the *SH3PX1* locus in our deletion mutants through CRISPR-mediated HDR (Fig. 7C,I; Fig. S3, Table S1).

In the absence of Nwk, there was an approximately 65% decrease in quantal content (Fig. 7D,I) (Coyle et al., 2004). To determine whether SH3PX1 acts in a pathway with Nwk to promote neurotransmitter release, we generated double mutants to assess the functional consequences of loss of both *nwk* and *SH3PX1*. If *SH3PX1* and *nwk* regulate synaptic vesicle exocytosis through distinct pathways, loss of both genes should result in an enhanced phenotype. If, by contrast, *SH3PX1* and *nwk* function in the same pathway, the loss of both would not be expected to yield a stronger phenotype than loss of *nwk* alone. Because changes in bouton number could complicate the interpretation of our results, we first assessed bouton number in the double mutants. *SH3PX1* does not modify the synaptic overgrowth phenotype observed in *nwk* single mutants (*nwk^2^*, *SH3PX1^10A^/nwk^2^*, *SH3PX1^C1^*: 33.3±0.98 boutons/NMJ 4, *n*=21 vs *nwk^2^*: 33.3±1.8 boutons/NMJ 4, *n*=11; NMJ 4; mean±s.e.m.). Consistent with the conclusion that the two proteins function in a common pathway to promote neurotransmitter release, we found that the quantal content deficits in *nwk* *SH3PX1* double mutants were not significantly different from that in *nwk* mutants (Fig. 7E,I). The observed trend towards lower release in double mutants might indicate that one or both proteins do not function exclusively in an SH3PX1–Nwk-dependent pathway to regulate neurotransmitter release.

Neurotransmitter release at the NMJ is regulated by factors intrinsic to the motor neuron, including proteins that regulate intracellular signaling, as well as retrograde signals that convey postsynaptic activity levels to modulate neuronal properties (Collins and DiAntonio, 2007; Harris and Littleton, 2015; Menon et al., 2013). To determine whether SH3PX1 functions in neurons, in the postsynaptic compartment, or both, we conducted tissue-specific rescue experiments. Neuronal, but not muscle, expression of full-length *SH3PX1* in null mutants restored quantal content to wild-type levels (Fig. 7F,I). This finding is consistent with the conserved physical interactions between SH3PX1 and Nwk, and indicates that SH3PX1 functions presynaptically to promote neurotransmission.

## DISCUSSION

Sorting nexins are key modulators of protein trafficking and intracellular signaling in diverse cellular contexts. We have identified conserved interactions between the BAR-SH3 subfamily of sorting nexins and Nervous wreck F-BAR proteins. Our loss-of-function studies in *Drosophila* reveal a new requirement for the sole BAR-SH3 sorting nexin, SH3PX1, in organizing presynaptic active zones and promoting neurotransmission. Double-mutant and tissue-specific-rescue experiments indicate that SH3PX1 functions presynaptically to promote neurotransmitter release and that it might act in Nwk-dependent and Nwk-independent pathways (Fig. 7). Ultrastructural analyses indicate that the baseline deficit in neurotransmitter release is unlikely to be explained by a primary effect on synaptic vesicles (Fig. 6). Instead, the substantial active zone abnormalities that we observed indicate a role for SH3PX1 in establishing functional presynaptic terminals.

Although our findings point to functional interactions between SH3PX1 and Nwk that promote neurotransmitter release, SH3PX1 does not function with Nwk to attenuate synaptic growth, suggesting that these Nwk-dependent roles are genetically separable and can be distinguished by SH3PX1 (Fig. 5). Genetic studies in *Drosophila* have shown that Nwk inhibits synaptic growth through the intracellular modulation of multiple growth factor signaling pathways (Koles and Budnik, 2012; Rodal et al., 2011), but how Nwk also promotes neurotransmitter release is not understood. One explanation is that impaired neurotransmitter release in *nwk* mutants is simply a secondary consequence of increased NMJ growth. However, Endophilin, which functions in a pathway with Nwk to regulate BMP signaling and the number of synapses, does not affect baseline evoked release, decoupling NMJ growth and function (Koh et al., 2004; Marie et al., 2004; Verstreken et al., 2002). Further, mutations in the BMP pathway, which result in significant synaptic undergrowth, yield functional impairments very similar to those observed in overgrown *nwk* mutants (Aberle et al., 2002; Marques et al., 2002; McCabe et al., 2004; Rawson et al., 2003). A potential mechanism for the divergent regulation of the establishment of synapse number and function comes from emerging evidence that muscle-derived BMP signaling regulates NMJ growth, whereas a distinct neuron-derived BMP signal promotes neurotransmitter release (James and Broihier, 2011; James et al., 2014). Finally, other crucial regulators of NMJ growth and function, including the E3 ubiquitin ligase Highwire and the B′ Protein Phosphatase 2A regulatory subunit Well-rounded, have been shown to perform these two roles through distinct pathways, although the pathways through which they regulate synaptic function have not yet been elucidated (Collins et al., 2006; Viquez et al., 2006).

How might SH3PX1 and Nwk interact to modulate a function-specific intracellular signal? The most detailed model of BAR-SH3 sorting nexin function comes from a large number of *in vitro* and cell culture studies of SNX9 (Daumke et al., 2014; Lundmark and Carlsson, 2009). During endocytosis, SNX9 binds to highly curved membranes at the neck of late clathrin-coated pits where it is thought to promote the scission of vesicles by recruiting dynamin and WASP (Lundmark and Carlsson, 2009). More recent evidence demonstrates that SNX9 levels peak following scission when SNX9 remains transiently associated with newly formed vesicles (Taylor et al., 2011). This raises the intriguing possibility that the later pool of SNX9 might act as a scaffold for proteins that are involved in the subsequent intracellular trafficking of cargo on newly formed vesicles to help dictate their fate.

Nwk localizes to recycling and early endosomes as well as to the plasma membrane of boutons, where its localization overlaps with that of SH3PX1, consistent with their conserved physical interaction (Figs 2–4) (Rodal et al., 2011). In mammalian cortical neurons, NWK2 and SNX9 both localize to presynaptic terminals, and NWK2 colocalizes with each member of the BAR-SH3 family at tubules induced by their overexpression (Figs 1 and 2) (Cao et al., 2013). BAR-SH3 sorting nexin interactions with Nwk are mediated by the sorting nexin SH3 domain and the Nwk F-BAR domain, suggesting that Nwk membrane-sensing activity might be inhibited in the bound state. A similar intramolecular interaction between the SH3 and F-BAR domains of Syndapin has been shown to inhibit membrane-deforming activity (Rao et al., 2010). Analogous intermolecular inhibitory interactions coupled with a temporal progression of binding partners that modulate inhibition could enable BAR-SH3 sorting nexins to act as plasma membrane scaffolds for intracellular proteins that have distinct membrane-sculpting properties, with the precise complement of the scaffolded complex determining or biasing the subsequent trafficking fate of associated cargo. In one possible model, SH3PX1 binding might act as a scaffold for specific Nwk–cargo complexes at late clathrin-coated pits while inhibiting Nwk F-BAR activity. On newly formed endosomes, SH3PX1 would rapidly dissociate, enabling Nwk to bind to and shape the early endosomal membrane, at which Nwk could participate in the formation and/or shuttling of specific cargo to a signaling or recycling compartment. Note that Nwk binds to both Dynamin and Wasp through its SH3 domains, and could play a role in recruiting both proteins in this scenario (Coyle et al., 2004; O'Connor-Giles et al., 2008; Rodal et al., 2008). Interestingly, an interaction between Nwk and another sorting nexin, Snx16, has previously been reported to direct cargo trafficking at the *Drosophila* NMJ (Rodal et al., 2011). Rodal and colleagues found that Nwk transiently interacts with Snx16, which contains a PX and coiled-coil domain and localizes to early endosomes, to attenuate growth factor signaling. Although this interaction occurs through a different molecular mechanism than that hypothesized above, it illustrates the importance of the interaction of Nwk with molecules that direct endosomal cargo sorting.

Our studies also suggest that SH3PX1 and Nwk promote neurotransmission through interactions with other proteins and pathways. In our mass spectrometry experiments, we identified a number of candidate NWK2-interacting proteins that are conserved in *Drosophila* and that might interact with Nwk to regulate synaptic function (Table 1). An intriguing candidate is Nipsnap, the single *Drosophila* homolog of a mammalian family of conserved vesicular proteins that perform multiple roles, including negative regulation of TRPV6 and L-type Ca^2+^ channel function (Brittain et al., 2012; Schoeber et al., 2008). It will be of interest to confirm the physical interaction through co-immunoprecipitation experiments and to generate null alleles to assess the functional interactions of Nipsnap in *Drosophila*. We also identified Spinophilin, a cytoplasmic scaffolding protein that has recently been shown to promote the accumulation of active zone components in *Caenorhabditis elegans* and *Drosophila* (Chia et al., 2012; Muhammad et al., 2015). We did not see the profound mislocalization of Bruchpilot that is observed in *Spinophilin* mutants in either *nwk* or *SH3PX1* mutants, although the ultrastructural defects in *SH3PX1* T-bars raise the possibility they could function at a common step. In addition, we identified two proteins with links to synaptic growth and/or maintenance: gelsolin, an actin-severing protein that promotes synapse elimination in worms and blocks neurogenesis in the mouse hippocampus (Kronenberg et al., 2010; Meng et al., 2015), and Hts/Adducin, an actin-spectrin-interacting protein whose loss in flies results in decreased synapse stability and the formation of small ectopic synapses (Pielage et al., 2011).

With their PX and BAR domains, BAR-SH3 sorting nexins can integrate information about membrane curvature as well as phosphoinositide content to play diverse roles in membrane trafficking. As such, members of the family have been linked to autophagy, macropinocytosis and phagocytosis, as well as clathrin-mediated endocytosis (Almendinger et al., 2011; Chen et al., 2013; Knaevelsrud et al., 2013a,b; Lu et al., 2011). Autophagy plays an important role in regulating synaptic growth at the *Drosophila* NMJ (Shen and Ganetzky, 2009). However, as synaptic growth was significantly impaired in all four autophagy mutants analyzed in that study, but not *SH3PX1* mutants, SH3PX1 does not appear to regulate autophagy at the NMJ. Knockdown of SNX9 with small interfering RNAs in cultured hippocampal neurons slowed synaptopHluorin fluorescence decay following a 30-s train of action potentials at 20 Hz, consistent with a role for SNX9 in synaptic vesicle recycling (Shin et al., 2007). Surprisingly, overexpression of Snx9 yielded a similar slowing of endocytosis, suggesting that the balance of endocytic proteins could be important for synaptic vesicle endocytosis. It will be of interest to determine whether SH3PX1, in addition to its role in establishing baseline transmission, has a role in synaptic vesicle recycling *in vivo*. Through its C-terminal SH3 and low-complexity domains, SNX9 interacts with the core endocytic and cytoskeletal proteins ACK2, AP-2, dynamin, synaptojanin and WASP (Lundmark and Carlsson, 2009). In *Drosophila*, SH3PX1 has been shown to bind to Ack, Wasp and Dock (Worby et al., 2002, 2001). As noted, Dynamin and Wasp function in a pathway with Nwk to regulate synaptic growth (Coyle et al., 2004; O'Connor-Giles et al., 2008; Rodal et al., 2008).

Future studies to identify additional functional interactors of SH3PX1 will be important to elucidate the Nwk-dependent, as well as any Nwk-independent, pathways through which SH3PX1 promotes the formation of an organized active zone and neurotransmitter release. These studies might also reveal interesting proteins that interact with postsynaptic SH3PX1. Although we have not yet identified postsynaptic defects in *SH3PX1* mutants through our morphological and functional analyses, in muscles, SH3PX1 is localized to the postsynaptic compartment where it could be involved in trans-synaptic communication with the presynaptic motor neuron, possibly in the context of synaptic plasticity. Our findings provide an *in vivo* model for elucidating the role of BAR-SH3 sorting nexins in directing the diverse intracellular signaling processes that regulate neural development and function.

## MATERIALS AND METHODS

### Cell culture

All mouse (*Mus musculus*) procedures were approved by the University of Wisconsin Committee on Animal Care and were in accordance with National Institutes of Health (NIH) guidelines. Swiss Webster animals of both sexes and stated ages were used. Cortical neuronal cultures were maintained and transfected, as previously described (Saengsawang et al., 2012). CAD cells (Qi et al., 1997) were grown in Dulbecco's modified Eagle's medium (DMEM)/HAMS F-12 supplemented with 10% FBS and 0.1 mg/ml penicillin-streptomycin. COS-7 cells (CRL-1651; American Type Culture Collection, Manassas, VA) were grown in DMEM, 10% FBS and 0.1 mg/ml penicillin-streptomycin. S2 cells (Schneider, 1972) were grown at 25°C in Schneider's medium supplemented with 10% FBS and 0.1 mg/ml penicillin-streptomycin. Transfections were performed using Lipofectamine2000 in CAD or COS7 cells, and Effectene in S2 cells per the manufacturer's instructions, and harvested 24–48 h post transfection.

### Antibodies

The following antibodies were used: mouse monoclonal anti-α-tubulin (western blotting, 1:40,000; Sigma-Aldrich #T9026), mouse monoclonal anti-BRP (immunofluorescence, 1:200; nc82, Erich Buchner, from the Developmental Studies Hybridoma Bank, Iowa City, IA), rabbit polyclonal anti-DsRed (western blotting, 1:1000; #632496, Clontech, Mountain View, CA), mouse monoclonal anti-FLAG (M2) (immunofluorescence, 1:500; western blotting, 1:4000; #F1804, Sigma-Aldrich, St Louis, MO), chicken polyclonal anti-GFP (western blotting, 1:5000, #13970 AbCam, Cambridge, UK), rabbit polyclonal anti-GFP (immunofluorescence, 1:1000, Life Technologies #A11122), rabbit anti-GluRIIC (immunofluorescence, 1:1000) (Marrus et al., 2004), monoclonal anti-hemagglutinin (HA) (western blotting, 1:3000; #3724, Cell Signaling Technologies, Danvers, MA), chicken polyclonal anti-NWK2 (western blotting, 1:1000; #6509, ProSci, Poway, CA), rabbit polyclonal anti-Nwk (immunofluorescence, 1:1000) (Coyle et al., 2004), rabbit anti-RIM-binding protein (immunofluorescence, 1:500) (Liu et al., 2011), rabbit polyclonal anti-SH3PX1 (immunofluorescence, 1:500; western blotting, 1:2000) (Hicks et al., 2015), mouse monoclonal anti-synapsin1 (immunofluorescence, 1:500; Synaptic Systems #106001) and rabbit anti-*Drosophila*-VGlut (immunofluorescence, 1:10,000) (Daniels et al., 2004). Species-specific Alexa-Fluor-488 and -568 secondary antibodies (Invitrogen, San Diego, CA) and anti-HRP-conjugated to FITC or Cy3 (Jackson ImmunoResearch, West Grove, PA) were used at a dilution of 1:500.

NWK1 antibody was raised in rabbit against a recombinant His-tagged N-terminal peptide corresponding to amino acid residues 653–764 (Cocalico Biologicals, Reamstown, PA). Briefly, the sequence coding for amino acid residues 653–764 was amplified from mouse cerebral cortex and cloned into the pET28c expression vector. Recombinant His-NWK1^653–764^ protein was expressed in BL21 cells, purified using Ni-NTA agarose and dialyzed against PBS. Following immunization in rabbits, antibodies were affinity purified using His-NWK1^653–764^ protein crosslinked to *CNBr*-activated *Sepharose 4 Fast Flow* following the manufacturer's protocol (GE Lifesciences, Marlborough, MA). Specificity was confirmed using siRNA against endogenous NWK1 in CAD cells (Fig. S4B).

### Protein interactions

#### Molecular reagents

Full length NWK1, NWK2 and SNX33 were amplified from mouse brain cDNA. SNX18 cDNA was synthesized (GENEWIZ, South Plainfield, NJ). SNX18 and SNX33 were cloned into ptdTomato and pEGFP vectors (Clontech, Mountain View, CA). pmCherry-Snx9 was generated by Christien Merrifield's laboratory (Taylor et al., 2011) and obtained from Addgene (Cambridge, MA). FLAG-tagged full-length *Drosophila* Nwk, FLAG–Nwk-F-BAR (amino acid residues 1–290), and HA–SH3PX1 were generated using the pAFW and pAHW expression vectors (*Drosophila* Gateway™ Vector Collection, generated by Terence Murphy and available through the *Drosophila* Genomics Resource Center, Bloomington, IN). All tags are N-terminal.

#### Mass spectrometry

FLAG–NWK2 was expressed in CAD cells through transient transfection. Cells were lysed in lysis buffer (50 mM Tris-HCl, pH 7.5, 150 mM NaCl, 1 mM EDTA, 1% Triton X-100 supplemented with a protease inhibitor cocktail) 48 h post transfection. Expression of FLAG–NWK2 protein was confirmed by immunoblotting with anti-FLAG monoclonal antibody M2. Lysates were cleared by centrifugation and immunoprecipitated with anti-FLAG-antibody-conjugated agarose. Beads were washed three times with FLAG-wash buffer (50 mM Tris-HCl, pH 7.5, 500 mM NaCl, 1 mM EDTA, 1% Triton X-100) and twice with TBS buffer (50 mM Tris-HCl, pH 7.5, 150 mM NaCl). FLAG-tagged protein was eluted with 100 μg/ml of 3×FLAG peptide in TBS buffer. An identical purification was performed with untransfected cells as control. The eluted fractions were subjected to trypsin digestion and tandem mass spectrometry (University of Wisconsin-Madison Biotechnology Center Mass Spectrometry/Proteomics Facility, Madison, WI).

#### Co-immunoprecipitation

COS-7 or S2 cells were transfected with tagged NWK1, NWK2, Nwk, SNX9, SNX18, SNX33 and/or SH3PX1 as indicated. Transfected cells were lysed in immunoprecipitation buffer (50 mM Tris-HCl pH 7.4, 150 mM NaCl, 1 mM EDTA, 1% Triton-X 100 and EDTA-free protease inhibitor cocktail) and centrifuged at 30,000 ***g***. Supernatants were incubated overnight at 4°C with 4 μg of the indicated antibody or control IgG, followed by incubation at 4°C for 2 h with Protein A/G agarose beads. Beads were washed extensively and resuspended in 1× SDS–PAGE loading buffer for western blot analysis. Each interaction was confirmed in at least three experiments.

### Immunofluorescence studies

Immunostaining in cortical neurons was performed as previously described (Saengsawang et al., 2012). COS-7 cells were fixed for 10–30 min in 4% formaldehyde in PBS at 24 h post transfection, washed three times in PBS and mounted in Vectashield. *Drosophila* larvae were obtained from population-controlled crosses of 10 females and 10 males reared at 25°C. Wandering third instar larvae were dissected in cold Ca^2+^-free saline followed by fixation with 4% formaldehyde in PBS for 10–40 min, depending on the antibody. Larvae were blocked in 1% BSA, followed by incubation with primary antibodies for 4 h at room temperature or overnight at 4°C and with secondary antibodies for 1–2 h at room temperature.

### Fly stocks

The following fly lines are available at the Bloomington *Drosophila* Stock Center (Bloomington, IN): *w^1118^,* Df(3L)BSC392 (BDSC-24416), *24B-Gal4*, *elav^c155^-Gal4*, *Act-Gal4*, *hs-Cre* and *vasa-Cas9.* UAS-FLAG-SH3PX1 flies were generated by cloning the full-length SH3PX1 cDNA into pBID-UASC-FG (Wang et al., 2012) and integrating into the attP2 landing site (Groth et al., 2004).

#### Generation of endogenously FLAG-tagged SH3PX1 flies

A 3×-FLAG tag and flexible linker sequence were integrated into a 1.8-kb genomic fragment flanking the *SH3PX1* start site (Fig. 4A). This fragment was cloned into pHD-DsRed to generate a donor template suitable for visible screening. A single nucleotide change was introduced to prevent CRISPR-mediated cleavage of the donor or engineered locus. The donor was co-injected with guide (g)RNAs targeting sites flanking the *SH3PX1* start site into *vasa-Cas9* embryos (Gratz et al., 2014). Following identification of engineered lines, the DsRed cassette was removed by crossing to *hs-Cre* flies. The final engineered lines were confirmed by molecular and western blot analysis (Fig. S4A). See Table S1 for all gRNA sequences used in this study.

#### Generation of *SH3PX1*-null alleles

*SH3PX1^10A^* and *SH3PX1^C1^* were generated by CRISPR-mediated HDR. Briefly, a single targeting gRNA (S1) and a donor plasmid containing 1-kb homology arms flanking a 3×P3-DsRed cassette were injected into *vasa-Cas9* embryos (Fig. S3, Table S1) (Gratz et al., 2014). Engineered lines were identified through DsRed expression in the eye and confirmed by molecular and western blot analysis (Fig. S4A).

#### Precise reversion of the *SH3PX1*-null allele

*SH3PX1^10A^* was repaired by CRISPR-mediated HDR (Fig. S3, Table S1). A donor template containing the genomic *SH3PX1* sequence was injected into *SH3PX1^10A^* mutants along with gRNAs targeting sites flanking the locus. *SH3PX1^repair^* flies in which the *SH3PX1* locus had been restored were identified through the loss of the DsRed marker, and confirmed through molecular and western blot analysis (Fig. S4A). *w^1118^; SH3PX1^repair^/+* flies were used as a control.

### Electrophysiology

Electrophysiological recordings were conducted using previously described methods (Bruckner et al., 2012). Briefly, third instar larvae were dissected in Ca^2+^-free HL-3 saline (70 mM NaCl, 5 mM KCl, 20 mM MgCl_2_, 10 mM NaHCO_3_, 115 mM sucrose, 5 mM trehalose, 5 mM HEPES, pH 7.2). Current clamp recordings from muscle six of segment A3 and A4 were performed in HL-3 containing 0.6 mM Ca^2+^. For each cell, 60 consecutive mEJPs were collected using pClamp software (Molecular Devices, Sunnyvale, CA), analyzed using MiniAnalysis (Synaptosoft, Decatur, GA), and averaged to obtain the mean amplitude and frequency. EJPs were evoked by applying the stimulus at 0.5-s intervals to the cut end of the appropriate segmental nerve. Stimulus amplitude was adjusted to recruit both 1s and 1b nerve inputs, and 100 consecutive EJPs were analyzed with pClamp software to determine the mean EJP amplitude for each NMJ. All cells included in the analysis had a mean resting potential between −60 and −80 mV, and an input resistance ≥5 MΩ.

### Imaging and morphological analyses

#### Confocal microscopy

Confocal images were acquired on an Olympus FV-1000 and processed using the Fiji distribution of ImageJ (Schindelin et al., 2012). The Adobe Photoshop auto-tone feature was used to linearly adjust brightness and contrast.

#### Electron microscopy

Samples were prepared for electron microscopy as previously described (Bruckner et al., 2012). Briefly, third instar larvae were dissected and fixed overnight in 2% paraformaldehyde and 2.5% glutaraldehyde in 0.1 M sodium cacodylate buffer at 4°C. Samples were washed in sodium cacodylate and postfixed in 2% osmium tetroxide for 1 h at room temperature, then dehydrated in ethanol, infiltrated and flat embedded in Epon resin. Trimmed blocks were sectioned on a LeicaEMUC6 ultramicrotome. Ultrathin (gray-silver) sections were collected on pioloform-coated copper slot grids and stained with uranyl acetate and Reynold's lead citrate. Images were collected on a Phillips CM120 transmission electron microscope and scored blind to genotype. The length of bouton and active zone membranes were traced in Fiji for measurement. A region encompassing all points within 200 nm of the active zone membrane was defined in Adobe Illustrator for quantification of synaptic vesicles.

### Statistical analyses

Single comparisons were conducted using Student's *t-*test or a Mann–Whitney test, following a D'Agostino-Pearson normality test. Welch's *t*-test used in cases of unequal variance. Multiple comparisons were performed using ANOVA followed by post-hoc tests with Šidák correction to maintain an experiment-wise significance level of 0.05. Chi-square tests were used to compare proportions.
